# A comparison of accommodation and ocular discomfort change according to display size of smart devices

**DOI:** 10.1186/s12886-020-01789-z

**Published:** 2021-01-18

**Authors:** Jeong Woo Kang, Yeoun Sook Chun, Nam Ju Moon

**Affiliations:** 1 Department of Ophthalmology, Airforce 16th Fighter Wing Medical Squadrone, Yecheon, Gyeongsangbuk-Do South Korea; 2 Department of Ophthalmology, College of Medicine, Chung-Ang University Hospital, 102 Heukseok-ro, Dongjak-gu, 06974 Seoul, South Korea

**Keywords:** Accommodation, Display size, Ocular discomfort, Smart device

## Abstract

**Background:**

To evaluate the change of accommodation and ocular discomfort according to the display size, using quantitative measurements of accommodation and ocular discomfort through subjective and objective metrics.

**Methods:**

Forty six subjects without any ophthalmic disease history were asked to watch the documentary movie, using two different sizes of smart devices; smartphones and tablets. Before and after using devices, the near point accommodation (NPA) and the near point convergence (NPC) were measured, and objective accommodation was measured using an auto refractometer/keratometer. The subjective ocular discomfort was assessed through a survey.

**Results:**

Both devices showed a decrease in post-use NPA and NPC, and the change after use of the smartphone was significantly severe, 1.8 and 2.5 folds respectively, compared to tablet (*p* = 0.044, *p* = 0.033, respectively). Neither smartphone nor tablet showed significant changes in the accommodative response induced by dynamic accommodative stimulus of auto refractometer/keratometer (*p* = 0.240 and *p* = 0.199, respectively). Subjects showed a more severe increase in ocular discomfort after using smartphones (*p* = 0.035) and reported feeling tired even with shorter use times (*p* = 0.012).

**Conclusions:**

Both devices showed significant decreases in NPA and NPC, and the larger changes were seen when using the small display smartphone. Even within 20minutes of using, subjects start to feel ocular discomfort, and it was more severe and faster after smartphones than tablets. Therefore, the smaller the display size, the greater the adverse impact on eyes, and thus, appropriate display size will need to be selected depending on the time and purpose of use.

## Background

With the drastic changes in the information age, the use of smart devices has risen. Such devices are convenient and easy to operate, and they allow end-users to perform diverse tasks, ranging from searching the internet to watching videos and for instantaneous messaging. According to data released by Statistics Korea in 2018, 89.6% of people over 3 years old are smartphone users. Since 2013, the use of smart devices has exceeded that of desktop devices. Among Korean smartphone users, 95.7% of them use their smartphones more than once a day, for an average of 10 hours and 47 minutes per week [1]. Furthermore, the proportion of smartphone use is increasing to the extent that 19.1% of smartphone users are classified as smartphone ‘addicts’, and mobile internet usage in the population aged over 60 years is also increasing [2].

As these smart devices become more crucial and integrated into our daily lives, we should consider how we are physically affected by them. The long-term use of smart devices is associated with visual and ocular symptoms, such as eye strain, blurring and dry and/or sore eyes. An increase in near field working hours with the use of smartphones can result in excessive accommodation, increasing ocular fatigue. In addition, continued use of smartphones reduces blinking, causing eye dryness [3]. For this reason, a growing number of patients are seeking clinical help due to their ophthalmic problems associated with increased use of smart devices.

Handheld smart devices are different from desktop or laptop computers in many aspects, such as the viewing position and distance, luminance, screen size, and usage patterns. In particular, the viewing distance for smart devices is relatively close compared to that of computers, which can result in eye strain due to accommodation and convergence [4]. Near-field work can cause constriction and accommodative spasms in the iris and ciliary muscle, which can lead to degraded accommodative functions [5]. This degradation of accommodative functions can have an adverse effect on ocular fatigue [5–8]. Research on the change in accommodation after the use of smartphones reported that accommodation was altered with smartphone and tablet use, with decreased amplitude and increased lag [9]. In terms of eye discomfort, blinking, tear function, and dry eye symptoms were compared before and after smartphone or tablet use. Smart devices that differ in size may have differential effects on the eyes, however, none of the studies compared changes with different display sizes.

Therefore, in our study, we investigated how the size of a smart device screen affects the user’s eye strain and ocular functions. To do so, we used objective metrics to perform quantitative measurements on changes in accommodation and eye discomfort after using smart devices of different display sizes.

## Methods

This prospective, comparative case series was approved by the institutional review board committee of the Chung-Ang University Hospital, Seoul, South Korea, and adheres to the tenets of the Declaration of Helsinki.

### Participant selection and study design

Participants were recruited on a volunteer basis using public notices. Among the participants, only those who had no history of ocular disease except correctable refractive error and were able to use smart devices were included in this study. We only included participants under 40 years old to exclude presbyopia. Participants with ophthalmic diseases (e.g., dry eyes, uncorrected refractive abnormality, ocular alignment disorder except heterophoria) or any diseases of the cornea, retina, or optic nerve were excluded of one’s will. Participants who had undergone operations other than refractive surgery were also excluded, and there were no restrictions related to astigmatism. After the purpose and design of this study were explained, each subject signed the informed consent form.

Subjects performed the tests using contact lenses or glasses to completely correct the refractive errors. Devices of two different sizes were used: the smaller device was the iPhone XR (Apple Inc., Cupertino, CA, USA) and the larger one was the iPad 9.7″ (Apple Inc.). The display sizes were 6.1 inches and 9.7 inches, respectively. Subjects were asked to watch a documentary video on the same platform (YouTube) for 1 hour. The same task was performed on each device in random order but on a different day more than a week later to avoid any carryover effects as a result of prior testing. When using a different kind of device, we used a different documentary video in random order to eliminate the possibility of a loss of concentration secondary to familiarity with the stimulus. To equalize conditions for near field working under the same conditions, the viewing distance at 30 cm and the maximum screen brightness were set at the normal indoor illumination level of 280 lux for the test.

### Visual function and self‐awareness symptoms

After the basic examination of visual acuity, intraocular pressure (IOP), and refractive error, vision correction was carried out using manifestation refraction. For the evaluation of accommodative functions, the near point of accommodation (NPA), near point of convergence (NPC), and accommodative responses to each stimulus at different distances were measured before and after using each smart device. For the subjective evaluation of NPA, the push-up method was used. The NPC was measured by approaching the eye strain with a 2 mm diameter circle to investigate the point at which one or both eyes diverged from fixation.

**Fig. 1:**
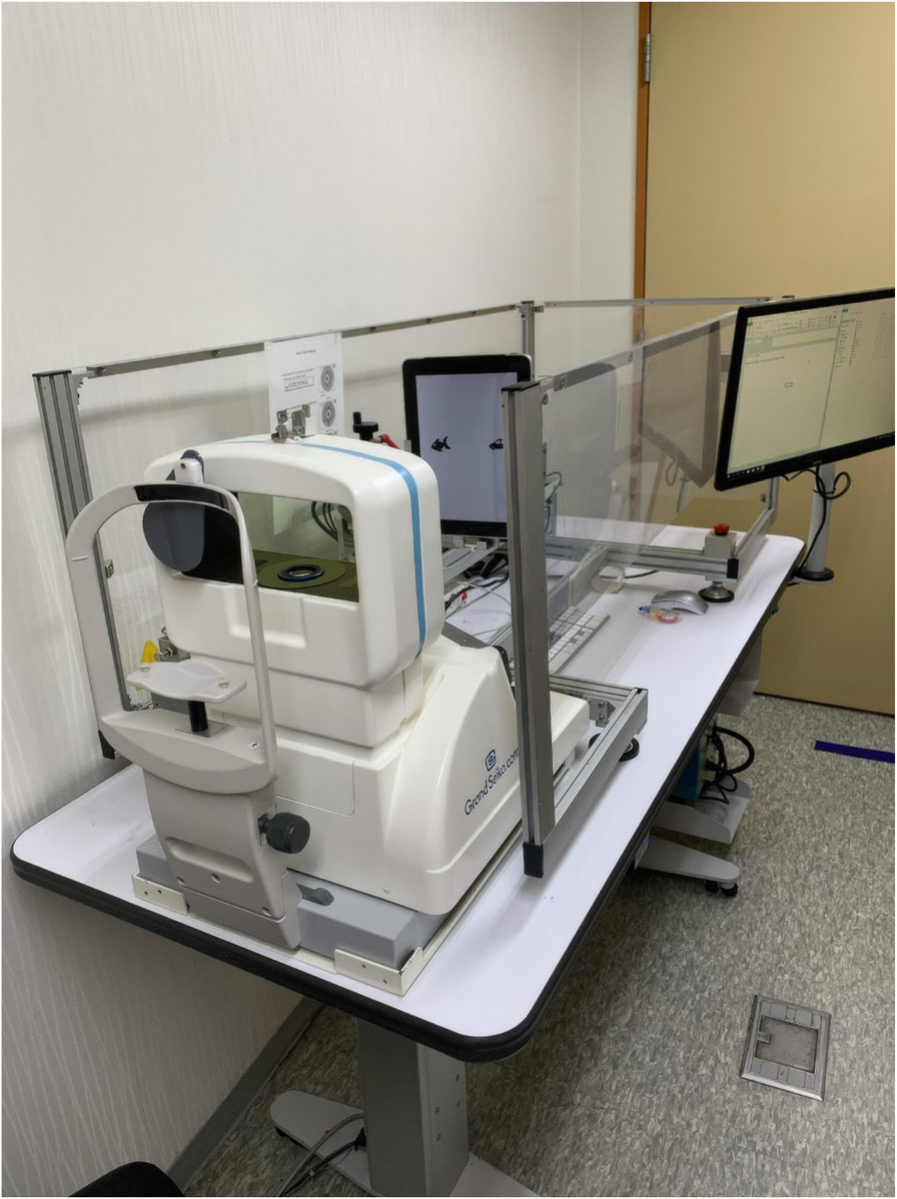
Measurement of objective accommodation using an autorefractometer/keratometer (WAM-5500, Grand Seiko, Tokyo, Japan). In the fully distance corrected state, one eye was covered. Next, a target was moved repeatedly at a constant speed at 33–100 cm from the uncovered eye to stimulate accommodation, and the autorefractometer continuously measured refraction based on the spherical equivalent

In addition to the above traditional static method, objective dynamic measurement of accommodation was conducted by the autorefractometer/keratometer (WAM-5500 Grand Seiko, Hiroshima, Japan; Fig. 1) under dynamic measurement mode. The measured spherical equivalent of the refractive error at one meter, 67 cm, and 33 cm was converted to an accommodation response. In this study, we compared accommodative lag, indicating insufficient response than accommodative stimulation, and accommodative facility, which is the flexibility to focus at a variety of viewing distances [10].

The pupil diameter was also measured using the same refractometer. Evaluation of stereopsis was conducted by Titmus Stereotest (Stereo Optical Co., Inc. Chicago, IL). Strabismus and heterophoria were measured using the alternate prism cover test, and tear film break-up time (tBUT) and the National Eye Institute (NEI) scale for grading corneal fluorescein staining were checked. The tBUT was measured using a fluorescein strip (Haag-Streit International, Koniz-Bern, Switzerland) to estimate the time at which a defect first occurred in the tear film. The NEI score, ranging from zero to 15, evaluates the corneal surface state by measuring fluorescein uptake [11].

**Fig. 2:**
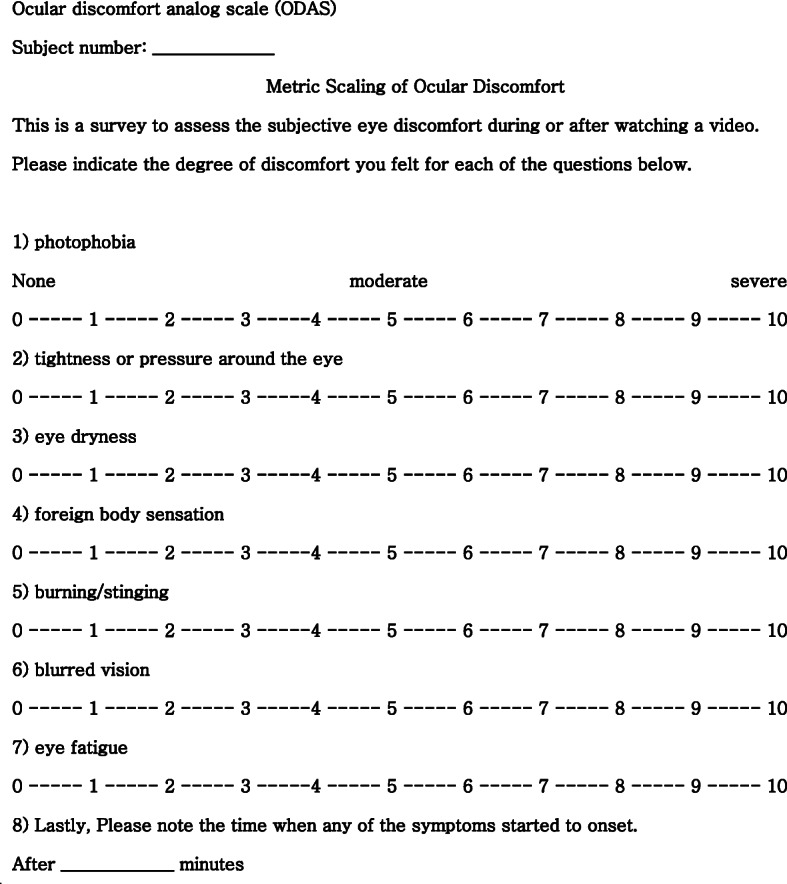
Ocular discomfort analog scale (ODAS). It consists of seven questions about the following symptoms: photophobia, tightness or pressure around the eye, eye dryness, foreign body sensation, burning or stinging, blurred vision, and ocular fatigue. Symptom severity was assessed on a scale from zero to 70, with scores of 0–10 for each category

Ocular discomfort was assessed using a validated Korean version of the Ocular Discomfort Analog Scale (ODAS) [12]. It is a self-report questionnaire modified from a questionnaire for assessing virtual reality viewing with a head-mounted display, and it is used as a measure of the level of ocular discomfort [13]. Subjects filled out the ODAS questionnaire using an analog scale after using each device. Moreover, we asked about the amount of time between the start of the video and the feeling of ocular discomfort. The computation of the score follows that which is outlined in Fig. 2.

All subjects underwent the same ophthalmic examinations twice, before and after using smart devices, and all tests were performed by the same ophthalmologist (J.W. Kang). Only data for the right eye were included in the analysis for all measurements except stereopsis, NPC, heterophoria, and ocular symptoms.

### Statistical analysis

Statistical analyses using SPSS version 20.0 (SPSS, Chicago, IL, USA). Differences in ocular discomfort, accommodation, and survey results between smartphone and tablet use were analyzed using the paired t-test. The results are expressed as mean ± standard deviation, and *p* values < 0.05 were considered statistically significant.

## Results

Overall, 46 subjects (22 men, 24 women) aged over 19 years old (ages ranged from 19 to 39 years, mean age 27.48 ± 5.95 years) were enrolled. The distance- and near-corrected visual acuity for all subjects was 20/20, and the average IOP was 15.28 ± 2.74 mmHg. The overall spherical equivalent of the converted refractive error was − 2.95 ± 2.31 diopters (D) (Table 1).

**Table 1:** Demographic characteristics of participants at baseline

Characteristics	N (%) or mean (± SD)
**No. of subjects**	46
**Age (yr)**	27.48 ± 5.95
**Sex (M : F no. (%))**	22(47.83%):24 (52.17%)
**Dominant eye (Right : Left (%))**	38(82.61%):8(17.39%)
**BCVA**	
**Far**	20/20
** Near**	20/20
**IOP (mmHg)**	15.28 ± 2.74
**Spherical equivalent (diopter)**	-2.95 ± 2.31
** Hypermetropia (+ 0.5D < SE, eyes)**	2
** Emmetropia (-0.5D < SE ≤ + 0.5D, eyes)**	7
** Myopia (SE ≤ -0.5D, eyes)**	37
** -3D < SE ≤ -0.5D**	12
** S.E ≤ -3D**	25

*BCVA* Best corrected visual acuity, *IOP* Intraocular pressure, *SE* Spherical equivalent, *D* Diopter

The baseline subjective accommodative power of NPA as estimated using the push-up method was 5.24 ± 0.77 cm. After using the smartphone and tablet, the post-NPA (increase in distance in cm) decreased to 5.43 ± 1.19 cm and 5.35 ± 1.01 cm, respectively. The NPA after smartphone use tended to decrease more than that after tablet use (*p* = 0.044). Baseline NPC was 10.22 ± 0.84 cm, and when using smart devices, it decreased to 10.46 ± 1.33 cm (smartphone) and 10.30 ± 1.09 (tablet). These changes were also statistically significant (*p* = 0.033; Table 2).

**Table 2:** Change of subjective static measurement of accommodation and other ocular parameters before and after use of smart devices

	Pre	Post Smartphone	*P* value^a^	Difference (Pre-Post)	*P* value^b^
		Tablet			
**NPA (cm)**	5.24 ± 0.77	5.43 ± 1.19	0.018	-0.20 ± 0.54	0.044
		5.35 ± 1.01	0.024	-0.11 ± 0.31	
**NPC (cm)**	10.22 ± 0.84	10.46 ± 1.33	0.010	-0.24 ± 0.60	0.033
		10.30 ± 1.09	0.044	-0.09 ± 0.28	
**Exophoria (PD)**					
** Far**	0.48 ± 1.39	0.48 ± 1.39	-	0	-
		0.48 ± 1.39	-	0	
** Near**	2.52 ± 3.90	2.26 ± 3.79	0.002	0.26 ± 0.53	0.160
		2.30 ± 3.79	0.006	0.22 ± 0.51	
** tBUT (sec)**	8.17 ± 3.63	7.83 ± 3.43	0.010	0.35 ± 0.87	0.299
		8.00 ± 3.58	0.198	0.17 ± 0.90	
** Stereopsis**	41.30 ± 3.41	41.96 ± 4.01	0.083	-0.65 ± 2.50	-
		41.74 ± 3.83	0.160	-0.43 ± 2.06	
** NEI score**	0.24 ± 0.64	0.30 ± 0.63	0.183	-0.07 ± 0.33	0.160
		0.22 ± 0.47	0.660	0.02 ± 0.33	
** IOP**	15.28 ± 2.73	15.50 ± 2.70	0.040	-0.22 ± 0.70	0.002
		14.89 ± 2.76	0.032	0.39 ± 1.20	
** Pupil diameter**	3.85 ± 0.89	3.88 ± 0.90	0.732	-0.03 ± 0.66	0.790
		3.91 ± 0.78	0.569	-0.06 ± 0.66	

*NPA* Near point accommodation, *NPC* Near point convergence, *PD* Prism diopter, *tBUT* Tear film break-up time, *NEI* National Eye Institute, *IOP* Intraocular pressure

^a^*p* value by paired t-test of comparison between pre-and post-test using smart devices

^b^*p* value by paired t-test of comparison between smart phone and tablet

The refractive responses to each stimulus in the objective evaluation of accommodation using an autorefractometer/keratometer are shown in Table 3. There was no significant change between before and after smart device use for either the smartphone or tablet. Accommodative facility, which is calculated by subtracting the values of the refractive response at 1 m and 33 cm to evaluate whether accommodation is properly regulated, was decreased more when a large display size was used, but it was not statistically significant (*p* = 0.847).

**Table 3:** Change of objective accommodative response before and after use of smart devices

	Pre	Post Smartphone	*P* value^a^	Difference (Pre–Post)	*P* value^b^
		Tablet			
**Refractive response (D)**					
** At 1 m**	-0.84 ± 0.40	-0.99 ± 0.68	0.103	0.15 ± 0.63	0.341
		-0.95 ± 0.76	0.232	0.11 ± 0.64	
** At 67 cm**	-1.64 ± 0.47	-1.72±-0.69	0.404	0.07 ± 0.58	0.002
		-1.58 ± 0.79	0.489	-0.06 ± 0.63	
** At 33 cm**	-2.07 ± 0.80	-2.12 ± 0.85	0.577	0.06 ± 0.68	0.363
		-2.07 ± 0.94	0.944	0.01 ± 0.63	
**Accommodative facility (D)**^**c**^	1.23 ± 0.55	1.13 ± 0.50	0.241	0.10 ± 0.55	0.847
		1.12 ± 0.57	0.199	0.11 ± 0.56	

*NPA* Near point of accommodation, *NPC* Near point of convergence

^a^*p* value by paired t-test of comparison between pre-and post-test using smart devices

^b^*p* value by paired t-test of comparison between smart phone and tablet

^c^ Accommodative facility = difference between refractive responses at 1 m − 33 cm

When the subgroup analysis of hypermetropic or myopic subjects was considered, there was no significant difference in the accommodation response depending on the size of the smart device display, except for the change in refractive response at 67 cm in the myopic group (Table 4).

**Table 4:** Change of accommodative response after use of smart devices by spherical equivalent

	Difference (Pre–Post)		*P* value^a^
	Post smartphone use	Post tablet use	
**Group 1: Hypermetropia or emmetropia (SE > -0.5 Diopter)**			
** NPA (cm)**	-0.33 ± 0.71	-0.22 ± 0.44	0.317
** NPC (cm)**	-0.78 ± 0.97	-0.22 ± 0.44	0.059
**Refractive response (D)**			
** At 1 m**	0.54 ± 1.32	0.33 ± 1.40	0.341
** At 67 cm**	0.39 ± 1.20	0.21 ± 1.31	0.263
** At 33 cm**	0.23 ± 0.95	0.10 ± 0.94	0.192
** Accommodative facility (D)**	0.31 ± 0.47	0.24 ± 0.65	0.767
**Group 2: Myopia (SE ≤ -0.5 Diopter)**			
** NPA (cm)**	-0.16 ± 0.50	-0.08 ± 0.28	0.083
** NPC (cm)**	-0.11 ± 0.39	-0.05 ± 0.23	0.324
**Refractive response (D)**			
** At 1 m**	0.06 ± 0.24	0.06 ± 0.25	0.942
** At 67 cm**	-0.01 ± 0.26	-0.13 ± 0.31	0.006
** At 33 cm**	0.12 ± 0.61	-0.02 ± 0.54	0.618
** Accommodative facility (D)**	0.04 ± 0.56	0.08 ± 0.54	0.560

^a^ *p* value by Wilcoxon signed rank test (Group 1), paired t-test (Group 2)

Changes in other ocular parameters are shown in Table 2. Distance exophoria did not change, but near exophoria shifted toward the ortho position after using both devices. The tBUT significantly decreased after using a smartphone (*p* = 0.010) but did not change after using the tablet (*p* = 0.198). Stereopsis decreased after use of both devices, but not significantly, and there was no significant change in NEI score. Intraocular pressure significantly increased after using a smartphone (*p* = 0.040), but was decreased after using a tablet (*p* = 0.032). Pupil diameter increased with use of both smart devices, but the change was not statistically significant.

For the survey of subjective symptoms of ocular discomfort, the total score increased following the use of each device (smartphone, 28.87 ± 9.88; tablet, 25.26 ± 13.84). Subjects reported that they felt less tired, in terms of ocular discomfort, when using a tablet than when using a smartphone. Furthermore, it took more time to feel fatigue using the tablet than the smartphone (*p* = 0.012; Table 5).

**Table 5:** Scores for subjective eye discomfort and time to fatigue onset

	Smartphone	Tablet	*P* value^a^
**Subjective eye discomfort**	28.87 ± 9.88	25.26 ± 13.84	0.035
**Time to fatigue (minutes)**	15.04 ± 6.60	17.83 ± 8.54	0.012

^a^*p* value by paired t-test

## Discussion

In typical studies of the ocular effect of smart devices, changes in accommodation and ocular discomfort before and after the use of smart devices, computers and/or paper, or the distance or usage time are compared. More recently, the display size of smart devices is an important factor in the smart device market, and tablet usage has grown due to its convenience. In other fields, the user’s perceived usability, effectiveness, and efficiency are actively studied according to the size of the display [14–17]. However, in ophthalmology, relatively little research is performed on the ocular effects according to the size of the smart device. Therefore, we investigated whether the change in accommodation or ocular discomfort differs depending on the size of the display.

Accommodation is the process of changing the refractive force to produce an image concentrated in the retina for different object distances. Normal binocular vision comprises vergence and accommodation systems that act simultaneously [18]. Because visual fatigue can result from vergence–accommodation conflict [19, 20], previous studies have evaluated visual fatigue objectively by using vergence and accommodation parameters, such as fusional vergence range, NPC, and the high-frequency component in microfluctuations of accommodation [21].

**Fig. 3:**
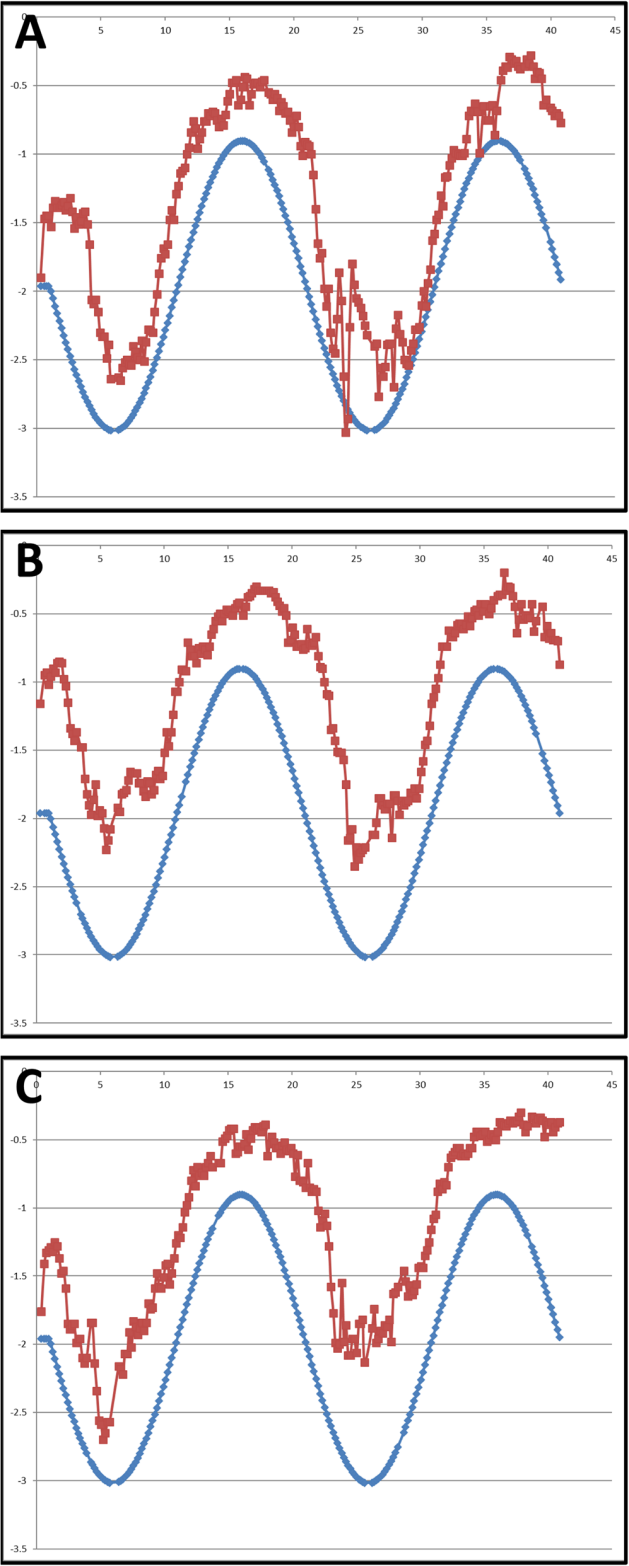
Example of accommodative responses to continuous stimulus measured by autorefractometer/keratometer. Accommodative responses are expressed as red dots to each stimulus (blue dots). Pre-test (**a**), Post-smartphone use (**b**), Post-tablet use (**c**)

Traditionally, NPA used to measure the amplitude of accommodation. However, participant-reported outcome measurements may lack objectivity and validity, and accuracy also remains an issue. In order to avoid such errors, many recent studies report evaluation of objective accommodation using the dynamic accommodometric function of the autorefractometer/keratometer. Win-Hall and Glasser [22, 23] used the WR-5100 K, an earlier model of the WAM-5500 used in their study, to measure objective accommodative control in young healthy people and presbyopic, phakic, and pseudophakic eyes. We used this newly developed dynamic method for the objective measurement of accommodation, so that the change of accommodative response after using smart devices is easily recognizable, as shown in Fig. 3.

In this study, NPA tended to decrease, and NPC showed a similar trend after use of smart devices. This tendency is consistent with the results of other studies in which NPA or NPC decreased after watching videos. The NPA was reduced by 3.8% after the use of smartphones and 2.1% after the use of tablets as compared to the pre-test measurement. Although the difference in display size, approximately 3.6 inches in diagonal length, was relatively small, the reduction in the subjective accommodative power was nearly two-fold. Likewise, NPC decreased 2.3% after the use of smartphones and 0.9% after the use of tablets, indicating that the change in accommodation is about 2.5 times more severe when viewing a small display.

Park et al. [24] reported that the decline in NPC after smartphone use was more larger than that after monitor use. Similarly, in our study, NPA and NPC were both aggravated after the use of smart devices. However, there was a difference in the amount of decrease between NPA and NPC. Ukai et al. [25] found that accommodation did not change significantly after viewing a movie for two hours. The relatively short one hour visual task used in this study may have had less effect on accommodation. Therefore, in this study, the time frame for the visual task may have been too short to determine any significant differences in NPC. However, these results clearly suggest that the smartphone display size may have an effect on changes in accommodation.

On the contrary, in the objective dynamic accommodation test, most accommodative responses after watching a video on either a smartphone or tablet were similar, and they even showed a tendency to increase compared to that before watching, even though there was no statistically significant difference. However, considering that the closest accommodative stimulation point of the autorefractometer/keratometer was limited to 33 cm, the maximum accommodation could have been underestimated. Moreover, there was not enough time for adequate accommodation at each distance, as the target of the autorefractometer/keratometer moved so rapidly in our setting, the measured responses may have been underestimated at every point. In addition, the examination was conducted immediately after viewing the screen at the near field for an hour, and a temporary myopic shift might have occurred due to residual accommodative spasms. Therefore, caution should be used when using only objective measurements, and the traditional subjective methods should be considered together to evaluate the accommodation more accurately.

In this study, phoria at far distances did not vary before and after the use of smart devices, but phoria at near distances tended to shift toward the ortho position. Furthermore, stereopsis decreased after using a tablet. This result is thought to be caused by vergence and accommodative adaptation. The examination to measure eye strain showed significant changes in IOP and tBUT after using smartphones. In particular, the increase in IOP when using a smartphone was significantly different from that when using a tablet. Ha et al. [26] demonstrated that working on a smartphone significantly increases IOP, and they postulate that it might be due to sustained active accommodation and convergence for near field work. Similarly, it is possible that the use of a small display smartphone caused thickening of the lens with excessive accommodation and an increase in IOP. Although the decrease in tBUT was not significant between the two devices, it was markedly reduced after using a smartphone, and may also be associated with the provocation of ocular discomfort.

After using a smart device, ODAS scores on the questionnaire showed an increase for all subjects, and users reported that they easily felt tired from the use of smart devices. Our results support previous evidence that ocular discomfort could be induced by visual tasks [27]. The difference between display sizes was also significant. When using a smartphone, participants felt more tired, and it took less time for them to feel ocular discomfort. On the other hand, it took longer to feel fatigued after using a tablet. In other words, people feel more intense discomfort within a shorter time with a relatively smaller display than with a larger display. These results may be because excessive accommodative convergence is necessary to form a clear image and the small size of the screen and displayed font induce visual fatigue.

There are several limitations of this study. First, the number of subjects was small and there was no limit on the degree of refractive error. Subsequent studies could overcome this vulnerability by recruitment of larger numbers of subjects with a pre-planned target of a certain number for each age group. Secondly, subjects were asked to watch a pre-determined specific video; however, individual concentration may have differed depending on the participants’ interests. Watching the video was a relatively passive visual task as compared to other tasks, such as reading a book. Such usage may therefore exert different effects on accommodation or ocular discomfort. Thirdly, the video viewing time in this study was short as 1 hour, which might have made it difficult to estimate changes in the eyes due to prolonged use. If smart devices are used for longer periods, the difference is likely to be greater. In addition, the difference in the display sizes of the smartphones and tablets used in the study may have been insufficient. We used the most recently released devices to reflect recent trends. However, as smartphones are larger with the addition of more diverse functions, the differences in the visual effects of the two devices may have been underestimated due to the small difference in display size. Finally, Various tests may be affected by subjective fatigue, and the subjects’ condition may have changed over the duration of the two visits.

In conclusion, accommodation change was more severe after the use of a smartphone with a relatively small display when compared to that after the use of a tablet with a larger display. And in terms of ocular discomfort, using smart devices with a smaller display size may provoke more discomfort. According to the results of this study, we suggest that the display size of smart devices can have different effects on the eye, so it should be adjusted according to the user’s accommodative functions and ocular discomfort when using smart devices.

## Data Availability

The datasets used and analyzed during the current study are available from the corresponding author on reasonable request.

## Abbreviations

NPA: Near point of accommodation
NPC: Near point of convergence
NEI: National Eye Institute
tBUT: Tear film break-up time
ODAS: Ocular discomfort analog scale
